# Improved Endothelial and Autonomic Function after Transcatheter Aortic Valve Implantation

**DOI:** 10.31083/j.rcm2405140

**Published:** 2023-05-08

**Authors:** Luka Vitez, Vito Starc, Borut Jug, Matjaž Bunc

**Affiliations:** ^1^Department of Cardiology, University Medical Centre Ljubljana, 1000 Ljubljana, Slovenia; ^2^Faculty of Medicine, University of Ljubljana, 1000 Ljubljana, Slovenia; ^3^Department of Vascular Diseases, University Medical Centre Ljubljana, 1000 Ljubljana, Slovenia

**Keywords:** aortic stenosis, transcatheter aortic valve implantation, endothelial function, autonomic function, flow mediated dilatation, heart rate variability

## Abstract

**Background:**

Degenerative aortic stenosis is an atherosclerotic-like process associated with impaired endothelial and autonomic function. Transcatheter aortic valve implantation (TAVI) has become a treatment of choice for patient with severe degenerative aortic stenosis at high surgical risk. The effect of this procedure on endothelial function measured with flow mediated dilatation (FMD) and autonomic function measured with heart rate variability (HRV) at different time-points of disease management (early and late follow-up) remains unknown.

**Methods:**

We prospectively included 50 patients with severe aortic stenosis who were deemed suitable for TAVI by the Heart Team. FMD and HRV parameters were collected at baseline (<24 h pre-TAVI), at early follow-up (up to 48 h post-TAVI) and at late follow-up (3–6 months post-TAVI).

**Results:**

43 patients (mean age 81 (75–85); 60% women) completed the study. FMD significantly improved from 2.8 ± 1.5% before TAVI to 4.7 ± 2.7% early after TAVI (*p *< 0.001) and was later maintained on late follow-up (4.8 ± 2.7%, *p* = 0.936). Conversely, high-resolution ECG parameters remained preserved at early and improved at late follow-up after TAVI. Significant improvement was detected in a high frequency-domain parameter—HF (from 5231 ± 1783 to 6507 ± 1789 ${\mathrm{ms}}^{2}$; *p* = 0.029) and in two Poincare plot parameters: ratio of the short- and long-term R-R variability in the Poincare plot—SD1/SD2 (from 0.682 to 0.884 ${\mathrm{ms}}^{2}$; *p* = 0.003) and short-term R-R variability in the Poincare plot—SDRR (from 9.6 to 23.9 ms; *p* = 0.001). Echocardiographic parameters comprising baseline maximal aortic valve velocity (R = 0.415; *p* = 0.011), mean aortic gradient (R = 0.373; *p* = 0.018), indexed stroke volume (R = 0.503; *p* = 0.006), change in aortic valve maximal velocity (R = 0.365; *p* = 0.031), change in mean aortic gradient (R = 0.394; *p* = 0.019) and NT-proBNP (R = 0.491; *p* = 0.001) were found as significant predictors of change in FMD.

**Conclusions:**

Endothelial function measured with FMD and autonomic function obtained with HRV parameters significantly improve after TAVI. While endothelial function improves early and is maintained later after TAVI, autonomic function remains stable and improves on late follow-up. This is most likely caused by early hemodynamic changes after resolution of aortic valve obstruction and gradual left ventricular remodeling.

**Clinical Trial Registration:**

www.clinicaltrials.gov, identifier NCT04286893.

## 1. Introduction

Degenerative aortic stenosis represents the leading native valve pathology in developed countries [1, 2]. Aortic valve degeneration is independently associated with same cardiovascular risk factors as coronary artery disease [3], showing us it is not only influenced by aging but also by an atherosclerotic-like processes including dynamic inflammation, lipid accumulation and calcification [4]. In the vasculature, all these structural alterations are preceded by endothelial dysfunction, a process of impaired vessel nitric oxide (NO) mediated regulation, present also in early stages of aortic stenosis [5]. Furthermore, it has recently been shown that atherosclerosis is associated with autonomic dysfunction [6], an imbalance between sympathetic and parasympathetic activity resulting in sympathetic predominance modulating heart rate response, cardiac contractility and vascular function [7]. It is influenced by intrinsic or extrinsic factors. Intrinsic factors are diseases that directly affect the autonomic nerves, such as diabetes mellitus and other neurological syndromes of primary autonomic failure. Extrinsic factors reflect changes that result as a consequence of cardiac diseases (e.g., myocardial infarction, heart failure, structural heart disease) [8]. The resulting sympathetic predominance generates a rise in catecholamines and inflammation cytokines that in turn, lead to worsening of heart failure, atherosclerosis, left ventricular hyperthrophy and increased risk of malignant arrhythmias [6, 8, 9]. Such sympathetic activity alterations have already been described in patients with degenerative aortic stenosis and connected with cardiovascular events and mortality [10, 11, 12, 13, 14].

In the last decade transcatheter aortic valve implantation (TAVI) became the treatment of choice for elderly patients at high surgical risk presenting with severe symptomatic aortic stenosis [15]. Previous studies have already demonstrated a significant improvement of endothelial function measured by flow mediated dilatation (FMD) at early and late follow-up after TAVI [16, 17, 18]. Conversely, studies on surgical valve treatment have yielded conflicting results suggesting a possible negative effect on endothelial function and subsequent early in-hospital recovery [18, 19, 20]. TAVI has also shown to have a lesser impact on autonomic function parameters measured by heart rate variability (HRV) in comparison to patients after surgical aortic valve replacement [21, 22]. Interestingly, its effects on long-term follow-up are still unknown. We hypothesized that improvement in hemodynamic proprieties after TAVI will have a positive effect on endothelial function which will be paralleled by improved autonomic parameters.

## 2. Materials and Methods

This prospective, single-center study carried out at the national TAVI referral University Medical Centre (UMC) Ljubljana, Slovenia, screened 50 consecutive patients eligible for TAVI between July 2019 and January 2020. Exclusion criteria were as follows: unstable cardiovascular disease or recent (<3 months prior to inclusion) cardiovascular events, acute illness or recent (<3 months prior to inclusion) non-cardiovascular disease requiring hospitalization, hemodynamic instability, stage 5 chronic kidney disease and active malignancy. The study was approved by the National Ethics Committee (reference number: 0120-215//2019/4) and performed in accordance with the ethical standards laid down in the 1964 Declaration of Helsinki and its later amendments. All participants sign an informed consent form prior to their inclusion. The study is registered in ClinicalTrial.gov (NCT04286893).

### 2.1 Endothelial Function

Endothelial function was assessed non-invasively measuring FMD at baseline pre-TAVI (<24 h prior procedure), early follow-up within 48 hours post-TAVI and late follow-up at 3 to 6 months. All vascular function measurements were assessed on the Aloka Prosound α7 ultrasound machine. FMD was measured on the right brachial artery and performed according to standardized practice by the same experienced investigator. Patients were fasted and abstained from coffee, smoking or exercise. The brachial artery was visualized horizontally approximately 1 to 2 cm above the antecubital fossa. 3 arterial diameter measurements were obtained ($d_{\text{baseline}}$) before inflating the cuff on the forearm with the pressure of 50 mmHg above patient’s systolic blood pressure. Limb ischemia was maintained for 270 seconds. 3 hyperemic brachial artery diameter measurements were obtained 60 seconds after cuff deflation ($d_{\text{hypaeremia}}$). FMD was later calculated as the percentage change in diameter using the following formula:

(1) $$\begin{array}{c} \mathrm{FMD}\left(\%\right)=\left[\left(\text{ mean }d_{\text{hypaeremia }}-\text{ mean }d_{\text{baseline }}\right)/\text{ mean }d_{\text{baseline }}\right]\times 100 \end{array}$$

Intra and interobserver variability were assessed on 20 healthy subjects. Intraclass correlation coefficient for FMD measurements was 0.95. Non-endothelial-dependent vasodilatation with nitroglycerin was not assessed due to safety concerns connected with hypotension in patients with severe aortic stenosis.

### 2.2 Autonomic Function

Autonomic function was evaluated based on HRV and its derived parameters. A 5-minute standard supine 12-lead ECG was recorded with a commercial computer-based ECG device (Cardiax, IMED, Budapest, Hungary). The recordings were analyzed using a custom software programme to calculate conventional and advanced ECG parameters [23, 24]. History or current atrial fibrillation and pacemaker implantation were exclusion criteria for further analysis. Patients were asked to lay still in a silent dark room while a 5-minute high-resolution ECG was recorded. After removing artefacts and arrhythmias (e.g., ectopic beats), we analyzed the listed time-domain measures: mean of all normal R-R intervals (Mean RR), standard deviation of all normal R-R intervals (SDNN), root mean square of successive R-R interval differences (rMSSD). This parameters quantify the amount of variability in measurements between successively recorded heartbeats. Additionally, we analyzed some frequency-domain parameters: logarithm of the total spectral frequency power of the Lomb periodogram (LO tot), low frequency power representing sympathetic activity (LF), high frequency power representing parasympathetic activity (HF), ratio of low and high frequency power representing the ratio between sympathetic and parasympathetic nervous system activity (LF/HF); and some non-linear parameters: ratio of the short- and long-term R-R variability in the Poincare plot (SD1/SD2), correlating with autonomic balance, and short-term R-R variability in the Poincare plot (SDRR), correlating with baroreflex sensitivity [25].

### 2.3 TAVI

TAVIs were performed in a high-volume (national referral) center by the same experienced operator. Valve and approach selection were left to the discretion of the local Heart Team.

### 2.4 Statistical Analysis

Our primary end-point was change of FMD at early and late follow-up. According to preliminary data a total of 31 patients would be required to detect a 1% FMD change (α = 0.05, β = 0.2). Accounting for a dropout rate of 10–20% in this elderly population we decided to include 50 patients. Baseline characteristics were described as mean values and standard deviations (normally distributed) or median and interquartile ranges (asymmetrically distributed) in case of continuous variables. Categorical variables were described as numbers and percentages. Comparison between means pre-TAVI and post-TAVI in the same group was determined with the paired sample t-test in case of normal and Wilcoxon U paired test for asymmetrical data distribution. Distribution was tested according to Shapiro-Wilk test. Repeated measurements ANOVA was used for comparison of continuous variables, using Bonferoni adjustment for post hoc analysis. Predictors of change in FMD and HRV parameters were calculated using the linear regression model – Pearson’s correlation coefficient. In a multivariate linear regression model, predictors for FMD change were assessed including age, sex and independent variables that emerged as single predictors (i.e., baseline maximal aortic valve velocity, mean aortic gradient, indexed stroke volume and NT-proBNP level). All data were analyzed using IBM SPSS Statistical v. 23 software (IBM Corp., Armonk, NY, USA) package with *p*-value of <0.05 considered statistically significant.

## 3. Results

After including 50 consecutive patients in the study, one was excluded due to acute illness, one had an unsuccessful TAVI procedure, one patient died and 4 (8%) were lost to follow-up due to COVID-19 restrictions accounting for a totaled drop-out rate of 15%. Mean age of 43 participants who completed the study was 81 (75–85), 26 (60%) were women (Table 1). All patients included in the study analysis had a successful TAVI implantation with CoreValve Evolut R or PRO (Medtronic, Minneapolis, USA) being the mostly implanted valves in 23 (53%) patients. One patient had a transaortic and one a valve-in-valve implantation. 6 (14%) patients received a pace-maker after TAVI. Aortic valve maximal velocity and mean gradient decreased from 4.3 ± 0.7 to 1.9 ± 0.4 m/s and from 45 ± 14 to 8 ± 4 mmHg respectively (*p *< 0.001). We also observed a significant reduction in non-invasively measured systolic pulmonary arterial pressure from 51 ± 14 to 43 ± 14 mmHg (*p* = 0.002).

**Table 1.** **Baseline characteristics of patients who completed the study**.

Baseline characteristics		Mean ± SD, median (Q1–Q3), n (%)
	Age, median (Q1-Q3), years	81 (75–85)
	Gender - female, n (%)	26 (60)
	BMI, mean ± SD, kg/$m^{2}$	27.2 ± 4.7
	Diabetes mellitus, n (%)	9 (21)
	Hypertension, n (%)	40 (93)
	Hyperlipidemia, n (%)	35 (81)
	Coronary artery disease, n (%)	22 (51)
	History of acute myocardial infarction, n (%)	7 (16)
	Peripheral artery disease, n (%)	3 (7)
	Carotid artery disease, n (%)	29 (67)
	History of cerebrovascular insult, n (%)	3 (7)
	Chronic obstructive pulmonary disease, n (%)	5 (12)
Medications		
	Aspirin, n (%)	23 (53)
	Oral anticoagulant, n (%)	17 (40)
	Angiotensin-converting enzyme inhibitor/angiotensin receptor blocker, n (%)	32 (74)
	Angiotensin receptor neprilysin inhibitor, n (%)	4 (9)
	Calcium channel blocker, n (%)	13 (30)
	Beta-blocker, n (%)	32 (74)
	Mineralocorticoid receptor antagonist, n (%)	8 (19)
	Furosemide, n (%)	29 (67)
	Statin, n (%)	30 (70)

Data are presented as number (%), mean ± SD or median (25th percentile–75th percentile).

**Fig. 1.**
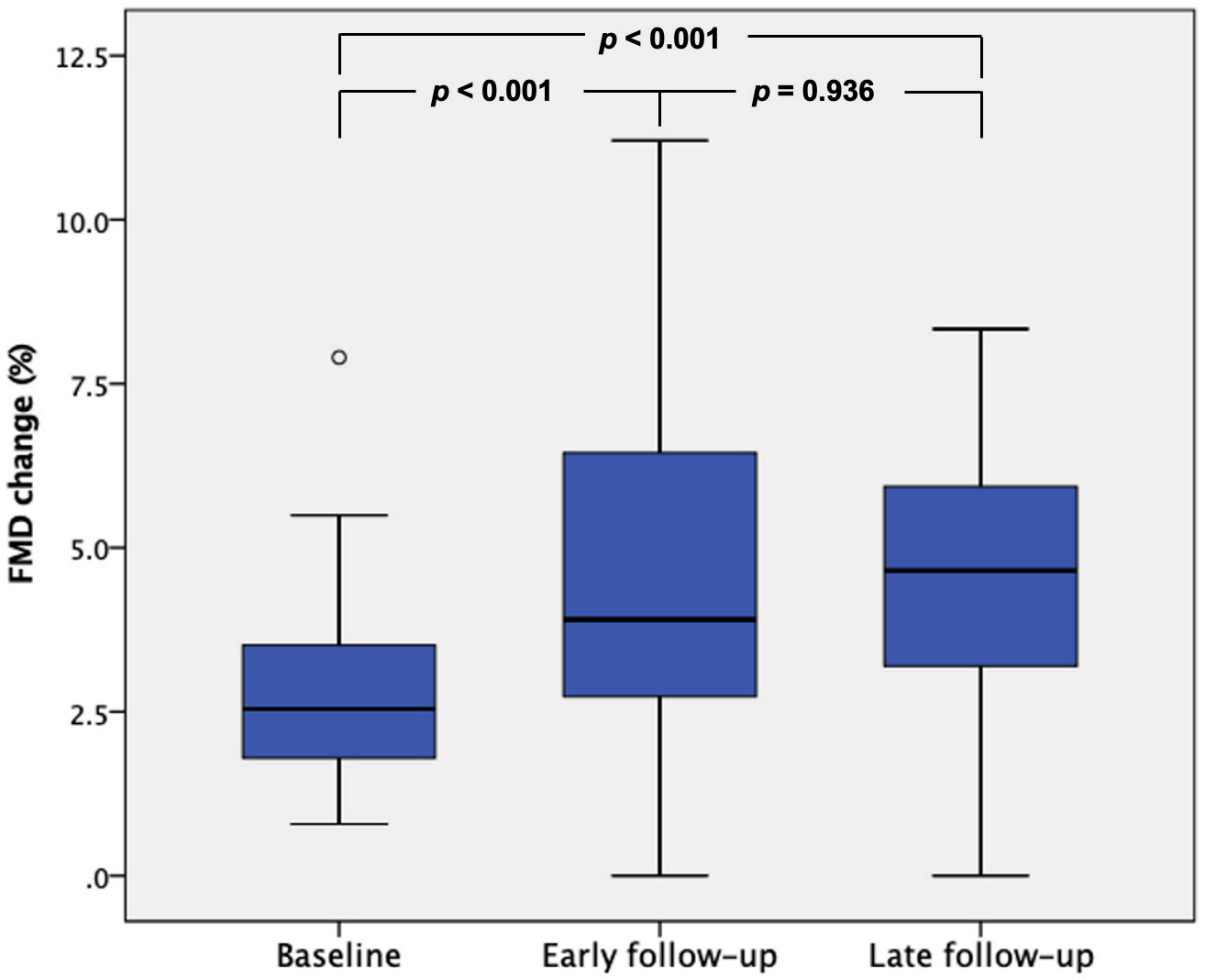
**Changes of mean FMD (%) on different follow-up times after TAVI**. FMD, flow mediated dilatation; TAVI, transcatheter aortic valve implantation.

FMD measurements improved significantly from 2.8 ± 1.5% before TAVI to 4.7 ± 2.7% early after TAVI (*p *< 0.001) and were later maintained on late 3–6 months follow-up with a FMD of 4.8 ± 2.7% (*p* = 0.936 when comparing with early follow-up results) (Table 2, Fig. 1). FMD differed significantly between time points (F (1.626, 63.418) = 9.063, *p *< 0.001). Measurements increased from baseline to early follow-up (–1.88 (95% CI, –3.01 to –0.75) %, *p *< 0.001), and from baseline to late follow-up (–2.0 (95% CI, –3.18 to –0.83) %, *p *< 0.001), but not from early to late follow-up (–0.12 (95% CI, –1.73 to 1.48) %, *p* = 1.0).

**Table 2.** **Changes in FMD and heart rate variability at baseline (pre-TAVI), on early follow-up (post-TAVI) and at 3–6 months late follow-up**.

	Baseline	Early follow-up	p*	Late follow-up	p*
FMD, mean (SD), %	2.8 (1.5)	4.7 (2.7)	<0.001	4.8 (2.7)	<0.001
Mean RR, mean (SD), ms	860 (119)	866 (147)	0.875	1002 (179)	0.064
SDNN, median (Q1–Q3), ms	50.2 (20.9– 76.9)	44.5 (18.7–62)	0.435	81.8 (35.4–115.2)	0.173
rMSSD, median (Q1–Q3), ms	50.8 (15.9–112.2)	55.4 (26.4–77)	0.287	114.8 (49.2–186.8)	0.087
LO tot, mean (SD), ${\mathrm{ms}}^{2}$	6187 (1681)	6076 (2116)	0.826	7146 (1594)	0.110
LF, mean (SD), ${\mathrm{ms}}^{2}$	4516 (2010)	4270 (2370)	0.629	5594 (1789)	0.129
HF, mean (SD), ${\mathrm{ms}}^{2}$	5231 (1783)	4764 (2702)	0.435	6507 (1789)	0.029
LF/HF, mean (SD)	0.86 (0.19)	0.89 (0.42)	0.724	0.84 (0.12)	0.545
SD1/SD2, median (Q1–Q3), ${\mathrm{ms}}^{2}$	0.682 (0.558–0.879)	0.676 (0.539–0.897)	0.678	0.884 (0.710–0.923)	0.003
SDRR, median (Q1–Q3), ms	9.6 (5.3–15.6)	13.5 (3.8–41.3)	0.653	23.9 (9.9–68.5)	0.001

Data are presented as mean (SD) or median (25th percentile–75th percentile). * = compared to baseline. FMD, flow mediated dilatation; Mean RR, mean of all normal R-R intervals; SDNN, standard deviation of all normal R-R intervals; rMSSD, root mean square of successive R-R interval differences; LO tot, logarithm of the total spectral frequency power of the Lomb periodogram; LF, low frequency power (sympathetic activity); HF, high frequency power (parasympathetic activity); LF/HF, ratio for sympatho-vagal balance; SD1/SD2, ratio of the short- and long-term R-R variability in the Poincare plot; SDRR, short-term R-R variability in the Poincare plot; TAVI, transcatheter aortic valve implantation.

When looking for predictors of change in FMD we found significant correlation with echocardiographic parameters comprising baseline maximal aortic valve velocity (R = 0.415; *p* = 0.011), mean aortic gradient (R = 0.373; *p* = 0.018), indexed stroke volume (R = 0.503; *p* = 0.006), change in aortic valve maximal velocity (R = 0.365; *p = *0.031) and change in mean aortic gradient (R = 0.394; *p* = 0.019). Furthermore, NT-proBNP levels before TAVI were also a significant predictor of change in FMD (R = 0.491; *p* = 0.001). Multiple linear regression modelling identified that younger age (B = –0.101, 95% CI: –0.198 to –0,004; *p* = 0.043) and lower baseline mean aortic gradient (B = –0.07, 95% CI: –0.135 to –0.005; *p* = 0.035) were associated with higher change in FMD.

**Fig. 2.**
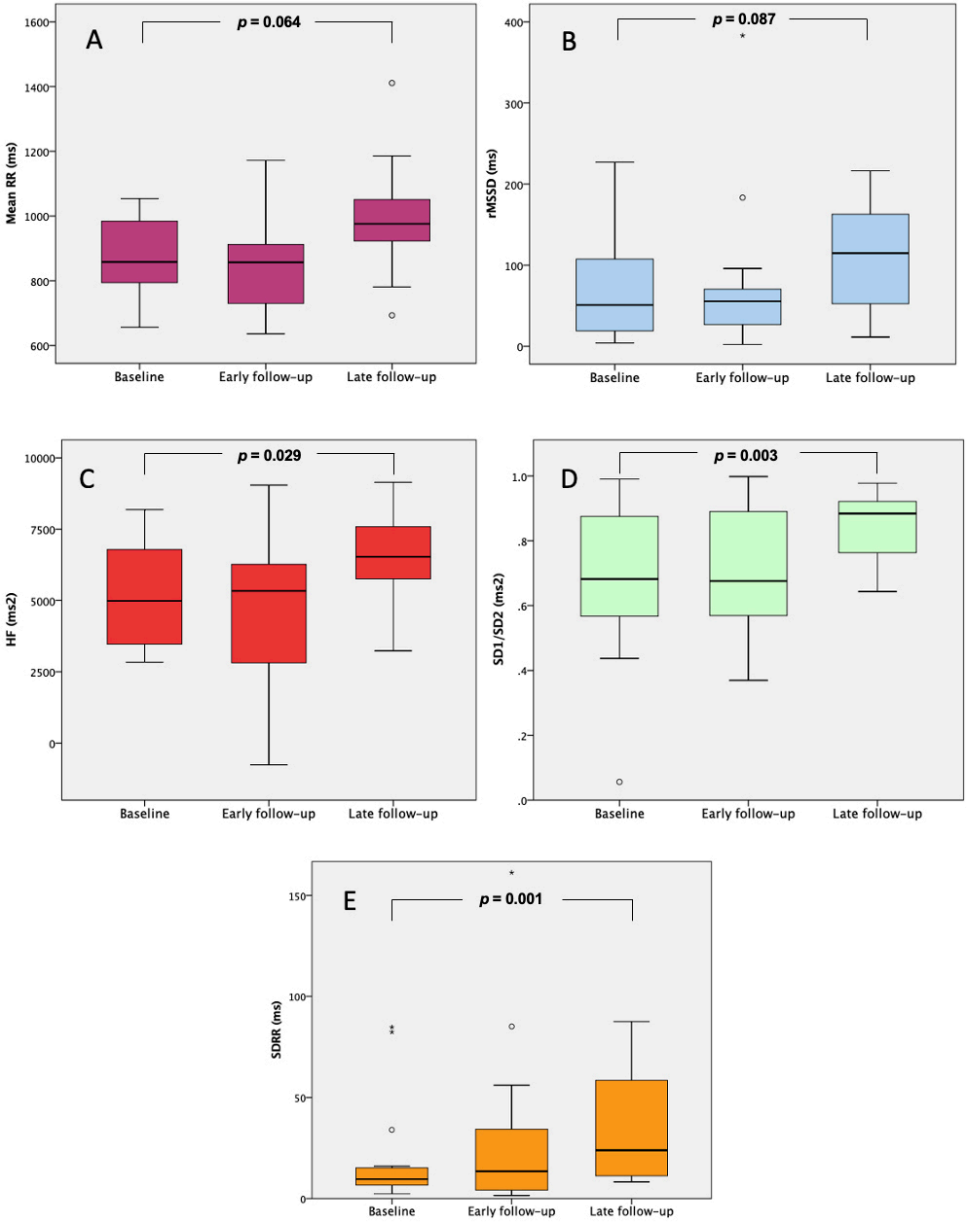
**Changes of HRV parameters on different follow-up times after TAVI (*p* values comparing baseline and late follow-up are shown)**. (A) Mean RR = mean of all normal R-R intervals. (B) rMSSD = root mean square of successive R-R interval differences. (C) HF = high frequency power (parasympathetic activity). (D) SD1/SD2 = ratio of the short- and long-term R-R variability in the Poincare plot. (E) SDRR = short-term R-R variability in the Poincare plot. HRV, heart rate variability; TAVI, transcatheter aortic valve implantation.

Autonomic cardiac functions measured by high-resolution ECG remained preserved at early and improved at late follow-up (3–6 months after TAVI). Significant improvement was detected in a high frequency-domain parameter-HF-representing parasympathetic activity (from 5231 ± 1783 to 6507 ± 1789 ${\mathrm{ms}}^{2}$; *p* = 0.029) and in two Poincare plot parameters: SD1/SD2 (from 0.682 to 0.884 ${\mathrm{ms}}^{2}$; *p* = 0.003) and SDRR (from 9.6 to 23.9 ms; *p =* 0.001). Two time-domain HRV parameters reached borderline significance: Mean RR increased from 860 to 1002 ms (*p* = 0.064) and rMSSD from 50.8 to 114.8 ms (*p* = 0.087) (Table 2, Fig. 2).

TAVI was also associated with significant decrease in hemoglobin levels after implantation from 126 ± 18 to 115 ± 18 mg/L (*p *< 0.001) with two reported serious bleeding events (one tamponade and one femoral access site bleeding). Changes in other laboratory results–including NT-proBNP, cholesterol levels, and liver and kidney function–were not statistically significant.

## 4. Discussion

Our study showed that endothelial function (measured with FMD) and autonomic function (obtained with high-resolution ECG-derived HRV parameters) significantly improved after TAVI. Improvement in vascular function was evident early after TAVI and remained relatively unchanged at late follow-up; conversely, selected indicators of autonomic function did not change immediately after TAVI, but increased only later on.

To the best of our knowledge, this is the first study investigating both endothelial and autonomic function after TAVI, at different time-points in the course of disease management. In patients undergoing TAVI, significant concomitant coronary artery disease can be found in up to 75% of cases [26]. Additionally, patients with degenerative aortic stenosis exhibit an atherosclerosis-like process paralleled by increased wall shear stress due to turbulent blood flow [5, 27, 28]. Laminar wall shear stress promotes endothelial cells survival and acts as a major determinant of endothelial apoptosis [29]. This activates a systemic adaptive mechanism with increased NO production and subsequent vasodilatation. The resulting basal hyperemic state impedes further vascular upregulation to stimuli, resulting in depleted NO reserves and impaired FMD [30]. This suggests that, after the resolution of aortic valve stenosis by non-invasive means of TAVI, wall shear stress rapidly decreases leading to reduced resting NO release and greater brachial vasodilatory response during FMD testing. Both FMD and cardiac autonomic function have been validated as predictors of prognosis in diverse populations, such as apparently healthy individuals, individuals with cardiovascular risk factors, patients with coronary artery disease and heart failure [31, 32, 33, 34, 35]. Our findings have shown these surrogate prognostic parameters improve in patients after TAVI, suggesting a systemic improvement in cardiovascular health not limited to the aortic valve intervention.

The early detected improvement in endothelial function could be explained by amelioration of hemodynamic proprieties immediately after TAVI. Post-TAVI hemodynamic recovery is characterized by an increase in ejection fraction and decrease in blood turbulence and wall shear stress [17, 36]. In our study, echocardiography-derived parameters of aortic stenosis hemodynamic severity (maximal aortic valve velocity, mean aortic gradient, change in aortic valve velocity and change in mean aortic gradient) emerged as significant predictors of FMD recovery immediately after TAVI. In addition, NT-proBNP levels before TAVI—i.e., a marker of increased ventricular wall stress from volume and pressure overload [37]—were also identified as an important predictor of FMD change. Interestingly, conventional cardiovascular risk factors (i.e., arterial hypertension, diabetes and hyperlipidemia) and coronary artery disease, otherwise associated with impaired endothelial function [38, 39, 40, 41], did not emerge as significant predictors of change in our patient population. Multiple regression modelling additionally identified that younger age and lower baseline mean aortic gradient result in higher change in FMD, suggesting treatment of severe aortic stenosis should be performed as early as possible when indications are fulfilled.

Previous studies have shown that different types of aortic valve interventions influence FMD differently. FMD seems to improve early after TAVI but transiently decrease after cardiac surgery [19, 20, 42], a characteristic most probably attributed to use of cardio-pulmonary bypass and its impact on systemic inflammation [42]. The latter translates into a vascular injury process caused by decreased NO bioavailability and hemolysis [43]. In contrast, transcatheter intervention seems to provide a quick and non-invasive approach to aortic valve physiological restitution and subsequent rapid patient recovery without affecting endothelial function.

HRV is a widely-used, non-invasive, indirect method for measuring cardiovascular autonomic regulation. Decreased HRV is linked to increased cardiovascular risk and mortality [44, 45]. Patients with degenerative aortic stenosis have a known sympatho-vagal imbalance with reduced HRV, potentially resulting in fatal arrhythmic complications [10, 12]. While surgery has been shown to further depress HRV on early follow-up, it remains stable after TAVI [21, 22]. The underlying mechanism has been mostly attributed to its non-invasive approach that avoids surgical heart manipulation, general anaesthesia and cardioplegia, direct surgical nerve damage during aortic clamping and incision, pain and potential surgical complications [21]. Our results support this finding with no significant change detected in both time and frequency-domain HRV parameters on early follow-up. However, in our study a potential early improvement in autonomic functions might have been counterbalanced by a significant 9% post-procedure reduction in hemoglobin levels. In fact, previous reports have demonstrated an association between anemia and decreased HRV in patients with coronary artery disease [46]. Importantly, we also observed a significant improvement in selected HRV parameters at late (3–6 months) follow-up. As cardiac hypertrophy of various etiologies including aortic stenosis has a known negative effect on HRV parameters [47], this delayed change might be attributable to gradual regression of left ventricular mass and left ventricular reversed remodeling after TAVI [48, 49]. The letter has already been described in a pilot study where morphological and functional changes where followed with advanced ECG-derived parameters [50].

In our study, both FMD and autonomic function parameters improved, albeit at different time points. On the one hand, vascular function seems to be more influenced by immediate (hemodynamic) effects of TAVI, while autonomic function may be more related to long-term effects, such as myocardial reverse remodeling. On the other hand, vascular and autonomic function can both be affected by a common underlying pathophysiology, such as chronic low-level inflammation [6, 51]. As such, the demonstrated paralleled improvement in FMD and HRV parameters might indicate that patients after TAVI experience a significant reduction in underlying patophysiologies, such as systemic inflammation and atherosclerosis progression. This gives us some more understanding into the demonstrated effectiveness of TAVI in elderly patients at high surgical risk [52, 53]. With the forthcoming advancement of TAVI indications to intermediate and low surgical risk groups more studies are needed to demonstrate its potential benefits in this study populations.

We have found some limitations in our study. First, this is a single-center observational pilot study and is therefore subject to its inherent methodological design, including unforeseen co-founders. Although UMC Ljubljana is the national reference center for TAVI patients and this population might be regarded as representative, larger multi-center international trials are needed to confirm our findings. Second, sample size is relatively small but powered enough to detect significant changes and comparable with previous studies observing FMD and HRV changes in patients with degenerative aortic stenosis [16, 17, 21, 22]. Third, FMD is an operator dependent ultrasound measurement technique with a great possibility of deviation in case of unexperienced personnel. Fourth, as the majority of this otherwise representative TAVI patient’s population were women and were treated with antihypertensive drugs this could have prevented a correct, independent analysis of autonomic functions in this study population. All data were collected by the same experienced operator in a center with extensive experience in performing FMD measurements [54, 55, 56].

## 5. Conclusions

Our data shows that endothelial and autonomic functions improve after TAVI. While endothelial function improves early and is maintained later after TAVI, autonomic function remains stable and improves on late follow-up. This is most likely caused by hemodynamic changes after resolution of aortic valve obstruction and gradual left ventricular remodeling. The overall improvement suggests a potential decrease in cardiovascular risk after TAVI in this study population. Our data needs to be interpreted with caution as it has been done on a relatively small sample.

## Data Availability

The data presented in this study are available on request from the corresponding author.
